# Tegoprazan-Amoxicillin Dual Therapy Versus Bismuth Quadruple Therapy Versus Tegoprazan-Based Quadruple Therapy for Helicobacter pylori Eradication: A Network Meta-Analysis

**DOI:** 10.7759/cureus.107000

**Published:** 2026-04-13

**Authors:** Roopa Velaga, Kinan Obeidat, Anastasia Postoev, Nawabzada Nadir Babar, Rahman Hameed Mohammed Abdul, Sonalben Chaudhary, Calvin R Wei, Danish Allahwala

**Affiliations:** 1 Internal Medicine, Jagadguru Jayadeva Murugarajendra Medical College, Davangere, IND; 2 Internal Medicine, University of Texas Medical Branch at Galveston, Galveston, USA; 3 Internal Medicine, Caribbean Medical University, Willemstad, CUW; 4 Internal Medicine, Combined Medical Hospital Lahore Medical College and Institute of Dentistry, Lahore, PAK; 5 Gastroenterology and Hepatology, Royal Derby Hospital, Stoke-on-Trent, GBR; 6 Internal Medicine, Zydus Sitapur Hospital, Sitapur, IND; 7 Research and Development, Shing Huei Group, Taipei, TWN; 8 Nephrology, Fatima Memorial Hospital, Karachi, PAK

**Keywords:** bismuth quadruple therapy, helicobacter pylori, network meta-analysis, potassium-competitive acid blocker, tegoprazan

## Abstract

*Helicobacter pylori* eradication remains a global clinical challenge, compounded by rising antibiotic resistance. Bismuth quadruple therapy (BQT) is the guideline-preferred first-line regimen, yet its high pill burden and adverse event profile limit real-world adherence. Tegoprazan, a novel potassium-competitive acid blocker (P-CAB), has demonstrated potent acid suppression and is being evaluated both as a dual therapy (tegoprazan-amoxicillin dual therapy (TADT)) and as a component of quadruple regimens (tegoprazan-based quadruple therapy (TBQT)). The aim of this study was to compare the efficacy, compliance, and adverse event profile of TADT, BQT, and TBQT for first-line *H. pylori* eradication using network meta-analysis (NMA) methodology. A systematic search of PubMed/MEDLINE, Embase, Cochrane Central Register of Controlled Trials (CENTRAL), Web of Science, and Scopus was conducted from inception to March 5, 2026. Randomised controlled trials (RCTs) and observational studies comparing at least two of the three specified regimens in treatment-naive adults were eligible. Eight studies (seven RCTs and one observational) encompassing 3,513 patients were included. All three regimens achieved statistically comparable *H. pylori *eradication rates. The risk ratio (RR) for BQT versus TADT was 0.82 (95% confidence interval (CI): 0.53-1.28), and for TBQT versus TADT, the RR was 1.01 (95% CI: 0.63-1.61). Treatment compliance was equally high across regimens (all comparisons p > 0.05). TADT was associated with a significantly lower risk of adverse events compared to BQT and TBQT. Global inconsistency testing confirmed model validity for all three outcomes (all p > 0.05). All three strategies yield equivalent *H. pylori* eradication efficacy and compliance as first-line treatment. TADT provides a significantly superior adverse event profile over BQT, making it a well-tolerated, simplified alternative suitable for most treatment-naive patients. TBQT offers comparable eradication with an intermediate safety profile and may be preferred in high antibiotic resistance settings. However, future large-scale RCTs are required to confirm these findings.

## Introduction and background

*Helicobacter pylori* (*H. pylori*) is one of the most prevalent chronic bacterial infections globally, affecting approximately 43% of the global population and serving as a major etiologic agent of gastrointestinal disorders, including peptic ulcers, gastric mucosa-associated lymphoid tissue lymphoma (MALT), and gastric adenocarcinoma [1,2]. Eradication of *H. pylori* remains a cornerstone strategy for reducing the incidence and progression of these complications, particularly gastric cancer.

Despite decades of clinical effort, optimising eradication therapy remains challenging. The escalating prevalence of antibiotic-resistant strains of *H. pylori* necessitates a change in management, as the conventional strategy of empiric-based treatments is becoming increasingly ineffective in both adult and paediatric populations, with eradication rates to common first-line regimens remaining suboptimal and continuing to decline [3]. Clarithromycin resistance, in particular, has rendered traditional triple therapy largely obsolete in many regions. In response, bismuth quadruple therapy (BQT) for 14 days is now the preferred regimen for treatment-naive patients when antibiotic susceptibility is unknown, according to the 2024 American College of Gastroenterology (ACG) guidelines. Optimised BQT, defined as a twice-daily proton pump inhibitor (PPI), bismuth salt at least 1,200 mg daily, metronidazole 1,500-2,000 mg daily, and tetracycline 2,000 mg daily, is recommended as the preferred first-line treatment for treatment-naive patients [4]. However, BQT carries a substantial pill burden, complex dosing schedules, and notable gastrointestinal adverse effects that can limit patient adherence.

The emergence of potassium-competitive acid blockers (P-CABs) has introduced new therapeutic possibilities. Tegoprazan, a novel P-CAB, offers a distinct pharmacological advantage over conventional PPIs through its rapid onset of action and more potent, sustained intragastric acid suppression, independent of meal timing and CYP2C19 polymorphism. Tegoprazan achieves a mean Tmax of approximately 1-2 hours, with peak intragastric pH elevation occurring within the first few hours of administration. Its bioavailability is not significantly affected by food intake, and it demonstrates a prolonged duration of acid suppression owing to its competitive, reversible binding to the H⁺/K⁺-ATPase pump. These pharmacokinetic properties support consistent acid control from the first dose, without the lag period associated with PPIs [5]. Potassium-competitive acid blockers have demonstrated enormous potential in the eradication treatment of *H. pylori* infection, with tegoprazan being one of the leading representatives [5]. Several regimens incorporating tegoprazan have been evaluated in recent trials. A 14-day course of tegoprazan-amoxicillin dual therapy (TADT) has been shown to be non-inferior to BQT for eradicating *H. pylori* [6], offering a simplified two-drug alternative. Simultaneously, tegoprazan-based quadruple therapy (TBQT), which incorporates bismuth alongside tegoprazan and antibiotics, has demonstrated promising eradication rates. Tegoprazan-based therapies achieved acceptable *H. pylori* eradication rates exceeding 85%, outperforming BQT [7], in a recent prospective trial from China. Furthermore, in clarithromycin-resistant *H. pylori* infection, tegoprazan was superior in efficacy to PPIs as first-line eradication therapy [8].

Despite these encouraging individual trial results, no network meta-analysis (NMA) has directly and simultaneously compared TADT, conventional BQT, and TBQT within a unified analytical framework. Existing meta-analyses have evaluated the efficacy and safety of P-CAB-based therapies compared to PPI-based therapies [9], but have not performed simultaneous indirect comparisons across these three specific regimens. This network meta-analysis therefore aims to synthesise all available evidence to determine the comparative efficacy, safety, and tolerability of TADT, BQT, and TBQT for the first-line eradication of *H. pylori*, thereby informing clinical decision-making in an era of escalating antibiotic resistance.

## Review

Methodology

This network meta-analysis was conducted and reported in accordance with the Preferred Reporting Items for Systematic Reviews and Meta-Analyses extension for Network Meta-Analysis (PRISMA-NMA) guidelines.

Literature Search and Search Strategy

A comprehensive and systematic search was conducted across major electronic databases: PubMed/MEDLINE, Embase, the Cochrane Central Register of Controlled Trials (CENTRAL), Web of Science, and Scopus. Databases were searched from inception to March 5, 2026. Additionally, the reference lists of all included studies, relevant systematic reviews, and clinical practice guidelines were manually screened to identify any further eligible studies not captured by the electronic search. ClinicalTrials.gov and the WHO International Clinical Trials Registry Platform (ICTRP) were also searched for ongoing or unpublished trials.

The search strategy was constructed using a combination of Medical Subject Headings (MeSH) terms and free-text keywords, adapted and tailored for each database. The search was organised around three principal conceptual blocks combined using Boolean operators. The population block incorporated terms such as "*Helicobacter pylori*", "*H. pylori*", and "*Campylobacter pylori*". The intervention and comparator block included terms such as "tegoprazan", "potassium-competitive acid blocker", "P-CAB", "amoxicillin", "dual therapy", "bismuth quadruple therapy", "bismuth-containing quadruple therapy", "quadruple therapy", and "tegoprazan-based quadruple therapy". The study design and outcome block incorporated terms relating to "randomised controlled trial", "RCT", "observational study", "prospective", "retrospective", "cohort", "eradication", "eradication rate", "adverse events", "side effects", "compliance", and "adherence". Within each conceptual block, synonymous and related terms were combined using the OR operator, whilst the three blocks were subsequently combined using the AND operator to yield a focused and comprehensive search. The full search strategy for each database is shown in Appendix A.

Eligibility Criteria

Eligibility criteria were defined prospectively and were structured according to the PICOS (Population, Intervention, Comparator, Outcome, and Study Design) framework. Regarding the study population, eligible studies were required to enrol adult patients aged 18 years or above with a confirmed diagnosis of *H. pylori* infection. With respect to interventions and comparators, studies were eligible if they compared at least two of the following three pre-specified treatment arms: TADT, consisting of tegoprazan combined with high-dose amoxicillin; BQT, comprising a PPI or P-CAB combined with a bismuth salt, a nitroimidazole or clarithromycin, and tetracycline or amoxicillin; or TBQT, defined as a regimen incorporating tegoprazan together with a bismuth compound and two antibiotics. Regarding study design, both randomised controlled trials (RCTs) and prospective or retrospective observational studies, including cohort and real-world evidence studies, were considered eligible for inclusion. Studies were required to report at least one of the pre-specified outcomes.

Studies were excluded if they exclusively enrolled paediatric populations below the age of 18 years, if they evaluated rescue or second-line salvage therapy in the absence of a treatment-naive arm, if they did not include any of the three specified comparator regimens, or if they were case reports, case series, editorials, narrative reviews, or conference abstracts lacking sufficient extractable data.

Study Selection

All records retrieved from the electronic database searches were imported into Rayyan systematic review management software (Rayyan Systems Inc., Cambridge, MA), and duplicate records were identified and removed prior to screening. Study selection was performed in two sequential stages by two independent reviewers. In the first stage, titles and abstracts of all retrieved records were screened independently against the predefined eligibility criteria, with records that clearly did not meet the inclusion criteria being excluded. In the second stage, the full texts of all potentially eligible records were retrieved and assessed independently by the same two reviewers. Any disagreements arising at either stage were resolved through structured discussion between the reviewers, and where consensus could not be reached, a third senior reviewer acted as an arbitrator whose decision was considered final.

Risk of Bias Assessment

The methodological quality of all included studies was assessed independently by two reviewers using validated, study design-specific instruments, with discrepancies resolved by discussion or third-party adjudication. For included randomised controlled trials, risk of bias was evaluated using the revised Cochrane Risk of Bias tool (RoB 2.0) [10], which examines five domains: bias arising from the randomisation process, bias due to deviations from the intended interventions, bias due to missing outcome data, bias in the measurement of the outcome, and bias in the selection of the reported result. Each domain and the overall study were classified as low risk, some concerns, or high risk of bias. For included observational studies, methodological quality was evaluated using the Newcastle-Ottawa Scale (NOS) [11].

Data Extraction

Data were extracted independently by two reviewers using a standardised, pre-piloted data extraction form developed in Microsoft Excel (Microsoft Corp., Redmond, WA) prior to commencement of the review. Any disagreements during data extraction were resolved through consensus discussion between the two reviewers, with recourse to a third reviewer where necessary. At the study level, the following variables were extracted from each eligible study: the first author's surname, the year of publication, the study design, the country or geographic region in which the study was conducted, the specific treatment groups evaluated, and the sample size per treatment arm. With respect to participant demographics, the mean age of enrolled patients, expressed either overall or per treatment arm as reported, and the number and proportion of male participants were extracted. Outcomes assessed in this study included the *H. pylori* eradication rate, adverse events of all grades, and treatment compliance.

Analysis Plan

The principal analytical method was a frequentist random effects network meta-analysis (NMA), which simultaneously compared all three treatment strategies (TADT, BQT, and TBQT) by jointly synthesising both direct and indirect evidence within a unified statistical framework. Unlike conventional pairwise meta-analysis, which is restricted to direct head-to-head comparisons between two interventions, the NMA framework enabled the integration of both direct evidence, derived from trials directly comparing two active regimens, and indirect evidence, estimated through a common comparator, thereby generating a comprehensive set of pooled estimates across all possible treatment pairs. This approach is particularly methodologically advantageous when more than two interventions are evaluated simultaneously and complete head-to-head evidence between all treatment pairs is unavailable. TADT was designated as the reference treatment throughout all analyses. For all outcomes, risk ratios (RR) were reported with 95% confidence intervals (CI). The consistency assumption, a fundamental prerequisite of NMA, asserting that direct and indirect estimates are in agreement, was formally tested using the node-splitting method and the design-by-treatment interaction model, with a p-value below 0.05 considered indicative of statistically significant inconsistency. The relative ranking of all three treatment strategies was determined using the Surface Under the Cumulative Ranking Curve (SUCRA), where higher SUCRA values indicate a greater probability of being the most efficacious or safest treatment option. All statistical analyses were performed using R software (R Foundation for Statistical Computing, Vienna, Austria), and a two-sided p-value of less than 0.05 was considered statistically significant throughout. As the number of studies were less than 10, we were unable to perform publication bias.

Results

**Figure 1:**
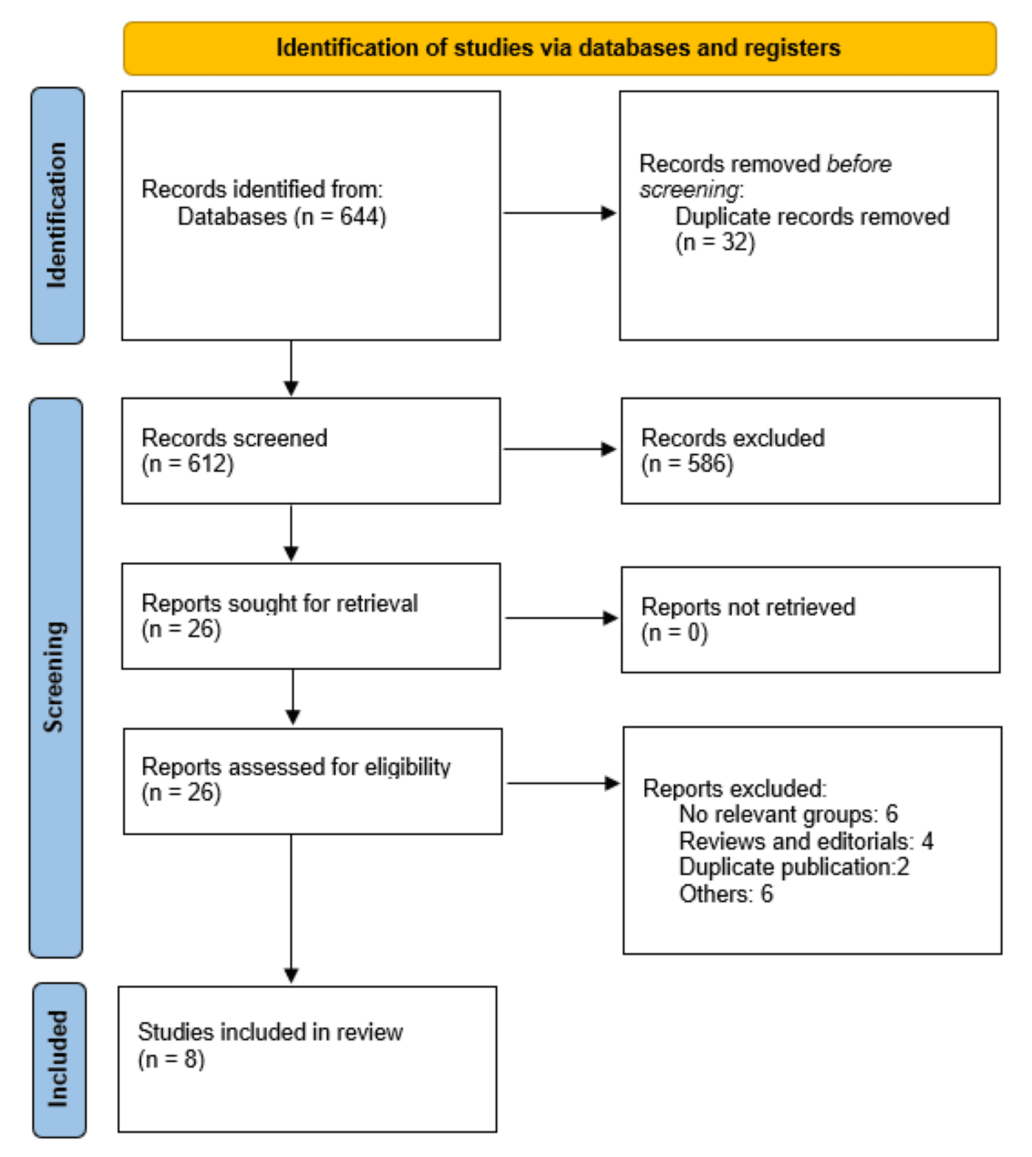
Study selection process

A total of 644 records were identified through systematic database searching across PubMed/MEDLINE, Embase, CENTRAL, Web of Science, and Scopus. Following deduplication, 612 unique records underwent title and abstract screening. Of these, 586 records were excluded on the basis of irrelevance to the research question, leaving 26 articles for full-text evaluation. After a detailed full-text assessment against the pre-specified eligibility criteria, eight studies were included in the final analysis. The complete selection process is illustrated in the PRISMA flow diagram (Figure 1).

Characteristics of Included Studies

**Figure 2:**
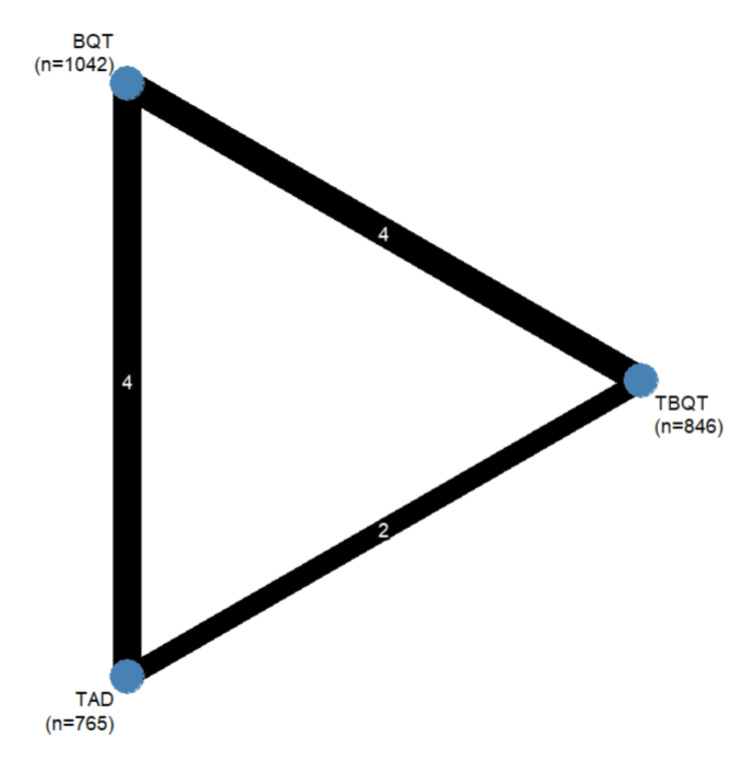
Network graph (eradication rate) Nodes represent individual treatment arms included in the network meta-analysis, with node size proportional to the total number of patients in each treatment group. The number in parentheses beneath each node label (n) denotes the total number of patients receiving that intervention across all included studies. Edges (connecting lines) represent direct head-to-head comparisons between two interventions, with line thickness proportional to the number of studies contributing to each comparison. The number displayed on each edge indicates the number of studies directly comparing the two connected interventions. BQT: bismuth quadruple therapy, TBQT: tegoprazan-based bismuth quadruple therapy, TAD: tegoprazan-amoxicillin dual therapy

**Figure 3:**
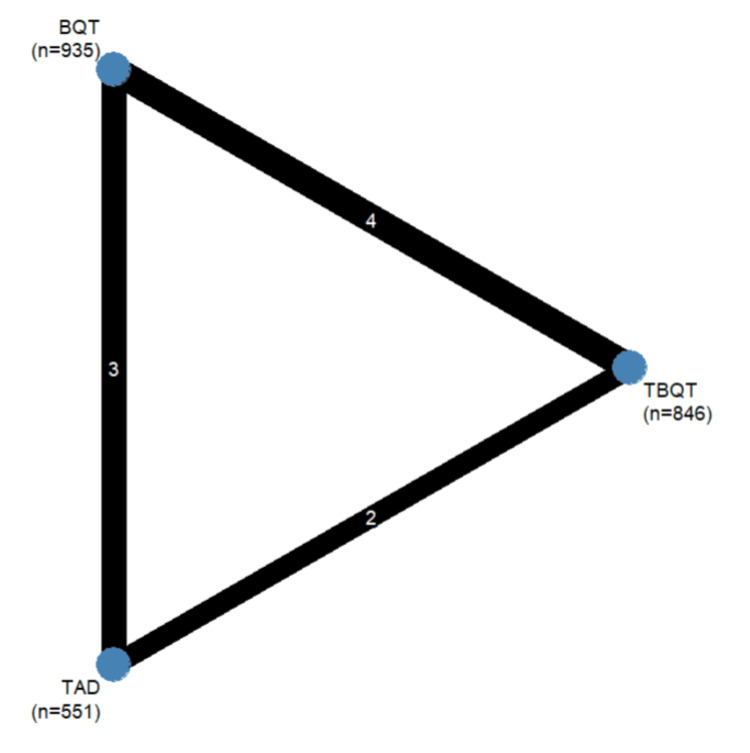
Network graph (compliance) Nodes represent individual treatment arms included in the network meta-analysis, with node size proportional to the total number of patients in each treatment group. The number in parentheses beneath each node label (n) denotes the total number of patients receiving that intervention across all included studies. Edges (connecting lines) represent direct head-to-head comparisons between two interventions, with line thickness proportional to the number of studies contributing to each comparison. The number displayed on each edge indicates the number of studies directly comparing the two connected interventions. BQT: bismuth quadruple therapy, TBQT: tegoprazan-based bismuth quadruple therapy, TAD: tegoprazan-amoxicillin dual therapy

**Figure 4:**
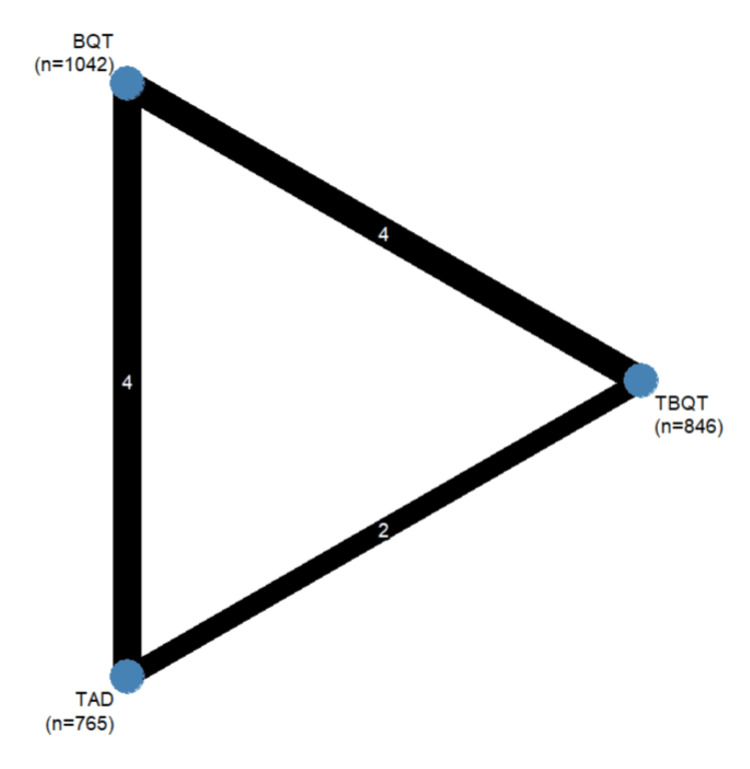
Network graph (adverse events) Nodes represent individual treatment arms included in the network meta-analysis, with node size proportional to the total number of patients in each treatment group. The number in parentheses beneath each node label (n) denotes the total number of patients receiving that intervention across all included studies. Edges (connecting lines) represent direct head-to-head comparisons between two interventions, with line thickness proportional to the number of studies contributing to each comparison. The number displayed on each edge indicates the number of studies directly comparing the two connected interventions. BQT: bismuth quadruple therapy, TBQT: tegoprazan-based bismuth quadruple therapy, TAD: tegoprazan-amoxicillin dual therapy

The eight included studies collectively enrolled 3,513 adult patients across treatment arms that included TADT, BQT, and TBQT. Seven of the eight studies were randomised controlled trials, and one was a retrospective observational cohort study. Geographically, six studies were conducted in China and two in South Korea. The detailed characteristics of all included studies are presented in Table 1. Table 2 and Table 3 present quality assessment of included RCTs and observational studies. Figures 2-4 present a network graph of all outcomes assessed in the meta-analysis.

**Table 1:** Characteristics of included RCTs RCT: randomized controlled trial, BQT: bismuth quadruple therapy, TBQT: tegoprazan-based bismuth quadruple therapy, TAD: tegoprazan-amoxicillin dual therapy, NR: not reported

Author ID	Study design	Region	Groups	Sample size	Mean age	Male (number)
Cheng et al. (2025) [6]	RCT	China	BQT	149	42	84
			TBQT	160	44.7	71
			TAD	159	44.1	59
Fan et al. (2025) [12]	RCT	China	TAD	130	NR	NR
			BQT	63		
Kim et al. (2022) [8]	RCT	Korea	BQT	105	57.9	59
			TBQT	106	58	61
Kim et al. (2021) [13]	Observational	Korea	BQT	188	55.7	102
			TBQT	193	55.3	102
Lin et al. (2024) [14]	RCT	China	BQT	107	NR	NR
			TAD	214		
Liu et al. (2025) [15]	RCT	China	TAD	118	26.2	103
			TBQT	118	27.3	103
Song et al. (2025) [16]	RCT	China	BQT	280	38.6	86
			TBQT	275	38.6	109
Yang et al. (2025) [17]	RCT	China	TAD	144	43.6	65
			BQT	144	41.27	54

**Table 2:** Quality assessment of included RCTs using the risk of bias in randomized trials (RoB 2) tool ● Low risk ◐ Some concerns (SC) ◯ High risk

Author, year	D1 Randomisation process	D2 Deviations from intended interventions	D3 Missing outcome data	D4 Outcome measurement	D5 Selection of reported result	Overall risk of bias
Cheng et al. (2025) [6]	● Low	◐ SC	● Low	● Low	● Low	◐ SC
Fan et al. (2025) [12]	● Low	◐ SC	● Low	● Low	● Low	◐ SC
Kim et al. (2022) [8]	● Low	● Low	● Low	● Low	● Low	● Low
Lin et al. (2024) [14]	● Low	◐ SC	● Low	● Low	● Low	◐ SC
Liu et al. (2025) [15]	● Low	◐ SC	● Low	● Low	● Low	◐ SC
Song et al. (2025) [16]	● Low	● Low	● Low	● Low	● Low	● Low
Yang et al. (2025) [17]	● Low	◐ SC	● Low	● Low	● Low	◐ SC

**Table 3:** Quality assessment of included observational study using the Newcastle-Ottawa Scale

Author ID	Selection	Comparison	Assessment	Overall
Kim et al. (2021) [13]	3	2	2	Good

Network Meta-Analysis: Eradication Rate

The NMA for *H. pylori* eradication rate incorporated data from all eight included studies, with TADT serving as the reference treatment. The evidence network comprised direct comparisons between TADT versus BQT (five studies) and TBQT versus BQT (four studies), with an indirect comparison between TADT and TBQT derived through the common BQT node.

**Figure 5:**
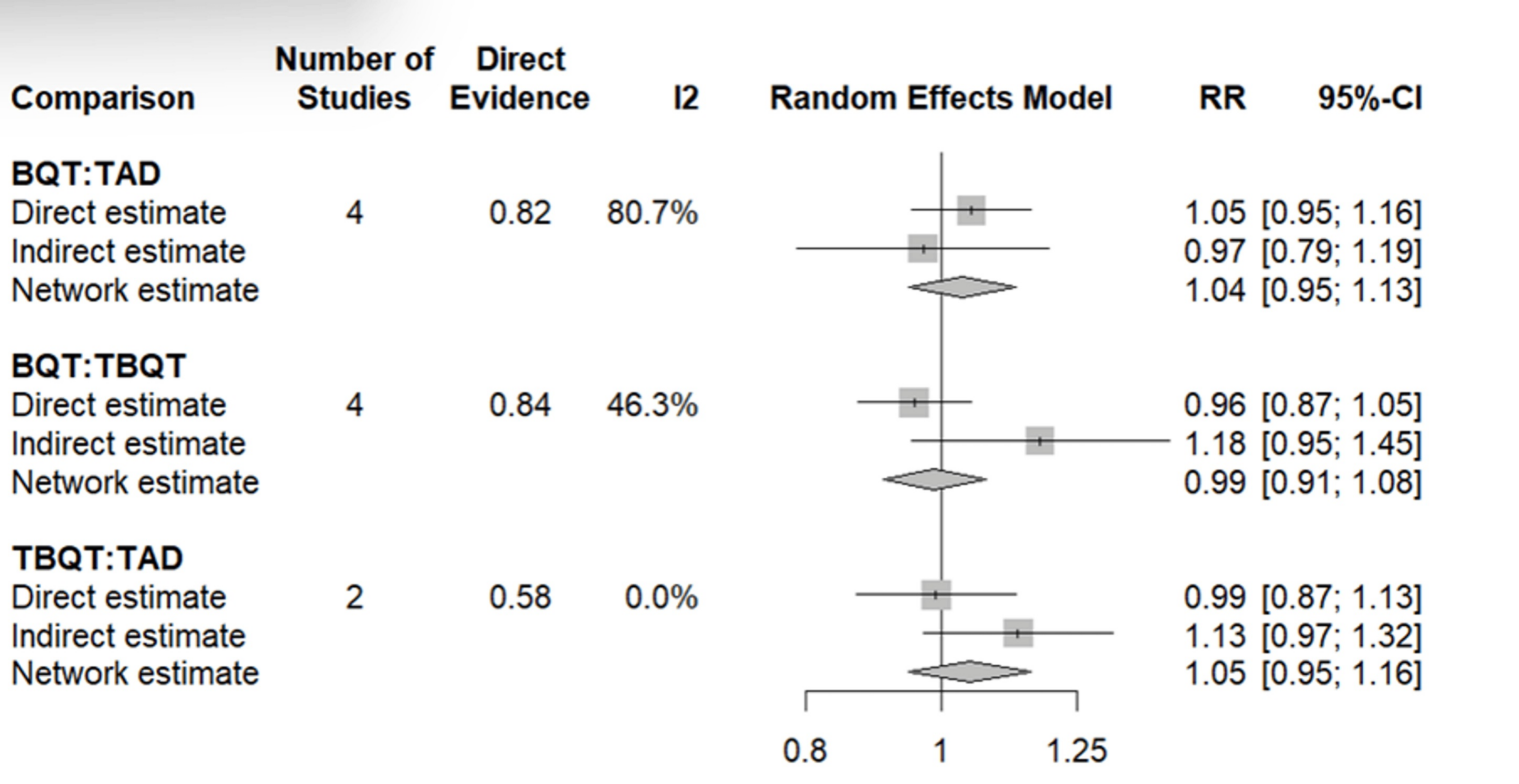
Network meta-analysis of eradication rate: pairwise comparisons between BQT, TBQT, and TAD Results are expressed as RR with 95% CI, with TAD as the reference comparator. RR > 1 indicates a higher probability of eradication rate compared to TAD. Each comparison presents three estimates derived from a frequentist random effects network meta-analysis. Direct estimate: the pooled effect derived solely from studies that directly compared the two treatments head-to-head, weighted by study size (represented by the size of the square) Indirect estimate: the effect inferred through a common comparator (i.e., via indirect chains in the evidence network), shown with a horizontal line and central point Network estimate: the combined estimate that integrates both direct and indirect evidence using the random effects model, represented by a diamond whose width reflects the 95% CI Heterogeneity across directly compared studies is quantified by the I² statistic. The direct evidence contribution to the network estimate is indicated by the Direct Evidence column. The vertical line at RR = 1 represents no difference between treatments. BQT: bismuth quadruple therapy, TBQT: tegoprazan-based quadruple therapy, TADT: tegoprazan-amoxicillin dual therapy, RR: risk ratio, CI: confidence interval

On pooled NMA, neither BQT nor TBQT demonstrated a statistically significant difference in eradication efficacy compared to TADT. The RR for BQT versus TADT was 0.82 (95% CI: 0.53-1.28), indicating that BQT was numerically associated with a lower probability of eradication; however, this difference did not reach statistical significance. Similarly, the RR for TBQT versus TADT was 1.01 (95% CI: 0.63-1.61), reflecting near-equivalent eradication performance between the two tegoprazan-based strategies. On SUCRA ranking, TBQT achieved the highest probability of being the most efficacious treatment (SUCRA = 83.2%), followed by TADT (SUCRA = 72.8%) and BQT (SUCRA = 56.0%), as shown in Table 4. The NMA forest plot for the eradication rate is presented in Figure 5.

**Table 4:** SUCRA score of outcomes assessed in this meta-analysis Given values are presented in percentages. Higher scores represented better progress. BQT: bismuth quadruple therapy, TBQT: tegoprazan-based quadruple therapy, TAD: tegoprazan-amoxicillin dual therapy, SUCRA: Surface Under the Cumulative Ranking Curve

Groups	Eradication	Compliance	Adverse events
TADT	72.8	74	100
BQT	56	54	19.8
TBQT	83.2	60	31.8

Network Meta-Analysis: Treatment Compliance

**Figure 6:**
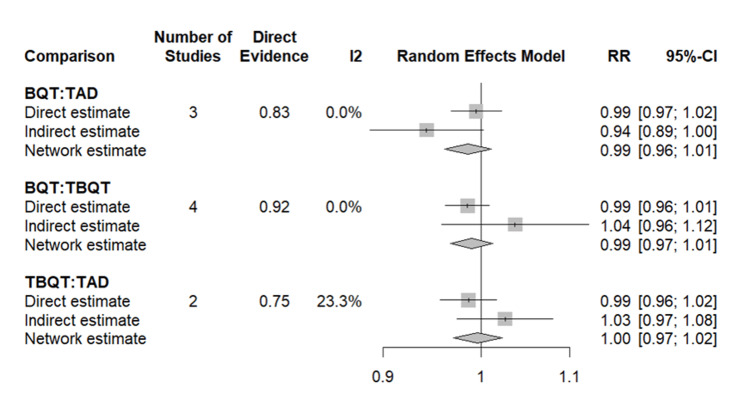
Network meta-analysis of compliance rate: pairwise comparisons between BQT, TBQT, and TAD Results are expressed as RR with 95% CI, with TAD as the reference comparator. RR > 1 indicates a higher probability of compliance compared to TAD. Each comparison presents three estimates derived from a frequentist random effects network meta-analysis. Direct estimate: the pooled effect derived solely from studies that directly compared the two treatments head-to-head, weighted by study size (represented by the size of the square) Indirect estimate: the effect inferred through a common comparator (i.e., via indirect chains in the evidence network), shown with a horizontal line and central point Network estimate: the combined estimate that integrates both direct and indirect evidence using the random effects model, represented by a diamond whose width reflects the 95% CI Heterogeneity across directly compared studies is quantified by the I² statistic. The direct evidence contribution to the network estimate is indicated by the Direct Evidence column. The vertical line at RR = 1 represents no difference between treatments. BQT: bismuth quadruple therapy, TBQT: tegoprazan-based quadruple therapy, TAD: tegoprazan-amoxicillin dual therapy, RR: risk ratio, CI: confidence interval

The NMA for treatment compliance incorporated data from seven of the eight included studies, with TADT as the reference. Pooled analysis demonstrated no statistically significant difference in compliance between any of the three treatment strategies. The RR for BQT versus TADT was 0.91 (95% CI: 0.52-1.59) and for TBQT versus TADT was 0.96 (95% CI: 0.55-1.68), both confirming broadly comparable adherence across regimens. SUCRA values further supported this conclusion, with all three treatments demonstrating similarly high probabilities of achieving good compliance, reflecting the consistently high adherence rates exceeding 90% observed across the included trials. No individual study identified a statistically significant between-group difference in compliance. The compliance NMA forest plot is presented in Figure 6.

Network Meta-Analysis: Adverse Events

**Figure 7:**
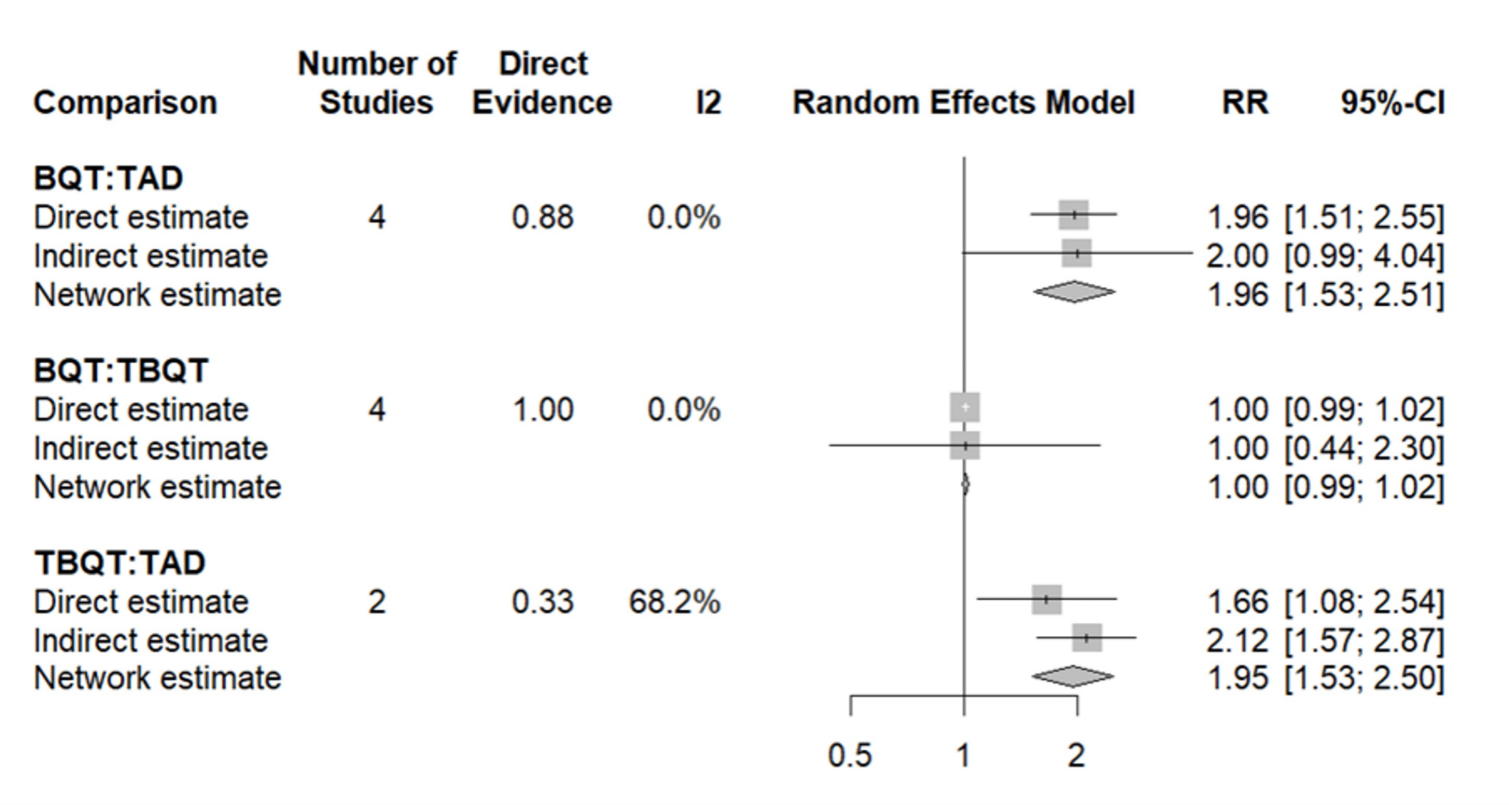
Network meta-analysis of adverse events: pairwise comparisons between BQT, TBQT, and TAD Results are expressed as RR with 95% CI, with TAD as the reference comparator. RR > 1 indicates a higher probability of adverse events compared to TAD. Each comparison presents three estimates derived from a frequentist random effects network meta-analysis. Direct estimate: the pooled effect derived solely from studies that directly compared the two treatments head-to-head, weighted by study size (represented by the size of the square) Indirect estimate: the effect inferred through a common comparator (i.e., via indirect chains in the evidence network), shown with a horizontal line and central point Network estimate: the combined estimate that integrates both direct and indirect evidence using the random effects model, represented by a diamond whose width reflects the 95% CI Heterogeneity across directly compared studies is quantified by the I² statistic. The direct evidence contribution to the network estimate is indicated by the Direct Evidence column. The vertical line at RR = 1 represents no difference between treatments. BQT: bismuth quadruple therapy, TBQT: tegoprazan-based quadruple therapy, TAD: tegoprazan-amoxicillin dual therapy, RR: risk ratio, CI: confidence interval

The NMA for adverse events of all grades was performed using TADT as the reference and incorporated data from all eight studies. In contrast to the eradication and compliance outcomes, a statistically significant difference between treatment groups was observed. TADT was associated with a significantly lower incidence of adverse events compared to BQT (RR: 0.42, 95% CI: 0.21-0.83), indicating that patients receiving TADT had substantially reduced odds of experiencing any treatment-emergent adverse event relative to those receiving BQT. The comparison between TBQT and TADT also favoured TADT (RR: 0.59, 95% CI: 0.30-1.17), although this difference narrowly missed conventional statistical significance. SUCRA values ranked TADT as the safest regimen (SUCRA = 100%), followed by TBQT (SUCRA = 31.8%) and BQT (SUCRA = 19.8%). The adverse events NMA forest plot is presented in Figure 7.

Consistency Testing

The consistency assumption was formally evaluated for all three outcomes using the global inconsistency test, and the results are presented in Table 5. No statistically significant inconsistency was detected for any outcome: the global test p-value was 0.23 for the eradication rate outcome, 0.3904 for treatment compliance, and 0.2610 for adverse events. As all p-values exceeded the threshold of 0.05, the null hypothesis of consistency between direct and indirect evidence could not be rejected, supporting the internal validity of all network estimates.

**Table 5:** Inconsistency assessment using design-based decomposition of Cochran's Q-statistic A significant p-value (p < 0.05) would indicate statistical inconsistency between direct and indirect evidence within the network. All p-values > 0.05 suggest no significant inconsistency across designs for any outcome.

Outcome	Q-statistics	P-value
Eradication rate	4.3	0.2311
Compliance	3.01	0.3934
Adverse events	4	0.26

Discussion

This network meta-analysis represents, to our knowledge, the first simultaneous indirect comparison of TADT, BQT, and TBQT within a unified analytical framework. By synthesising data from eight studies encompassing 3,513 patients, we demonstrate three principal findings: first, that all three regimens achieve statistically comparable* H. pylori* eradication efficacy; second, that treatment compliance is equivalently high across all three strategies; and third, that TADT is associated with a significantly lower burden of adverse events compared to BQT, with TBQT occupying an intermediate position. These findings carry meaningful implications for clinical practice, particularly in the context of escalating antibiotic resistance and growing emphasis on patient-centred care.

The absence of a statistically significant difference in eradication rates among TADT, BQT, and TBQT is the central finding of this analysis. Conventionally, BQT has been endorsed as the preferred empirical first-line regimen in international guidelines, including the 2024 ACG Clinical Guideline, due to its established efficacy across regions with variable antibiotic resistance profiles [2]. This recommendation is supported by robust evidence demonstrating that 14-day BQT achieves intention-to-treat (ITT) eradication rates approaching 90% [18]. Our NMA confirms that BQT remains an effective strategy, but critically establishes that both TADT and TBQT are capable of matching this performance.

The equivalence of TADT to BQT in our analysis aligns with and extends the findings of several recent RCTs. Kong et al. demonstrated that 14-day TADT was non-inferior to esomeprazole-amoxicillin dual therapy in the SHARE2301 trial, with ITT eradication rates of 85.8% versus 84.2%, respectively [3]. Fan et al. similarly reported that 14-day TADT was non-inferior to BQT with an ITT eradication rate of 73.7% versus 75.0%, whilst critically demonstrating that treatment duration matters, as the 10-day TADT arm was significantly inferior to both [12]. These trial-level findings are translated into network-level evidence by our NMA, confirming that 14-day TADT is a clinically equivalent alternative to BQT for first-line eradication.

The comparable performance of TBQT and TADT is biologically plausible. Tegoprazan's potassium-competitive mechanism affords more rapid, sustained, and CYP2C19-independent acid suppression than conventional PPIs [19]. When this more potent acid blockade is combined with bismuth and antibiotics in TBQT, the resulting intragastric pH environment is theoretically optimised for antibiotic activity. Kim et al. (2022) found that tegoprazan-based bismuth quadruple therapy was non-inferior to lansoprazole-based BQT, with per-protocol eradication rates of 90.2% versus 82.4%, respectively [8], suggesting that substituting PPIs with tegoprazan within a quadruple framework may yield a modest numerical benefit. Whilst TBQT ranked numerically highest on SUCRA for eradication, the credible intervals for all pairwise comparisons overlapped substantially. SUCRA rankings should therefore be interpreted with caution in the presence of wide credible intervals, as they do not imply statistically significant superiority [20]. The practical implication is that treatment selection may reasonably be guided by tolerability, pill burden, local resistance patterns, and availability rather than efficacy alone.

The most clinically actionable finding of this analysis is the significantly lower adverse event burden associated with TADT relative to BQT (RR: 0.42, 95% CI: 0.21-0.83). The adverse event profile of BQT is well-characterised and predominantly gastrointestinal in nature, encompassing nausea, taste disturbance, abdominal pain, and diarrhoea, driven by the multi-antibiotic component of the regimen, particularly tetracycline and metronidazole [2]. By contrast, TADT contains only amoxicillin as an antibiotic alongside tegoprazan, substantially reducing the pharmacological source of antibiotic-related toxicity.

This finding parallels evidence from the vonoprazan-amoxicillin dual therapy literature. Multiple studies of vonoprazan-based dual therapy, a pharmacologically analogous regimen, have consistently demonstrated lower adverse event rates versus BQT, with one Chinese RCT reporting overall adverse event rates of 11.2% versus 20.8% in favour of dual therapy [21]. The present NMA extends this class-level safety signal specifically to tegoprazan-based dual therapy. Treatment-emergent adverse events are a recognised driver of medication non-adherence in *H. pylori* eradication therapy, and inadequate compliance is one of the strongest independent predictors of eradication failure [22]. By minimising adverse events, TADT may indirectly reinforce treatment completion and thereby sustain its eradication efficacy in real-world settings where adherence is imperfect.

The position of TBQT in the adverse event hierarchy is also noteworthy. Although TBQT was numerically associated with fewer adverse events than BQT, the difference did not reach statistical significance, and TBQT ranked between TADT and BQT on SUCRA. This suggests that replacing PPIs with tegoprazan within a quadruple framework may attenuate but does not eliminate the adverse event burden attributable to the multi-antibiotic component. This is consistent with the broader meta-analytic evidence that tegoprazan-based regimens are associated with fewer mild adverse events compared to PPI-based equivalents, particularly with 14-day durations [7].

All three regimens achieved high and statistically comparable compliance rates in this NMA. This finding is reassuring from a public health perspective and suggests that none of the three strategies poses a prohibitive adherence barrier in the context of supervised clinical trials. However, real-world adherence to a four-drug, multi-dosing BQT regimen may be substantially lower than that observed in trial settings, as the logistical complexity of taking three or four medications at multiple daily time points presents a genuine practical challenge for many patients [18]. The simpler two-drug TADT regimen, therefore, retains a theoretical real-world adherence advantage that may become more apparent in pragmatic studies.

Implications for Clinical Practice and Guidelines

The findings of this NMA support a re-evaluation of the clinical positioning of tegoprazan-based regimens in contemporary *H. pylori* eradication guidelines. The 2024 ACG Clinical Guideline already recognises P-CAB-amoxicillin dual therapy as a suitable empiric alternative to BQT for treatment-naive patients without penicillin allergy [2], a recommendation now substantiated at the network level by our analysis. Our NMA provides confirmatory network-level evidence that 14-day TADT is eradicationally equivalent to BQT whilst being significantly better tolerated, strengthening the evidence base for its clinical adoption.

From an antibiotic stewardship perspective, TADT offers additional advantages. It requires only two agents, reduces total antibiotic exposure by eliminating metronidazole and tetracycline, and may be particularly well-suited to regions with high metronidazole or tetracycline resistance where BQT efficacy may be compromised [23]. Furthermore, amoxicillin resistance among *H. pylori* strains remains globally low, typically below 5%-10%, making it a durable backbone for dual therapy strategies [24,25]. TBQT occupies a distinct clinical niche: whilst its efficacy is comparable to both TADT and BQT and its safety profile numerically superior to PPI-based BQT, it retains a four-drug framework that may be most appropriate in high-resistance settings or in patients who have previously failed simpler dual therapy.

Regarding optimal dosing, the included studies employed variable tegoprazan dosing schedules, most commonly 50 mg twice daily (bd) or three times daily (tid), in combination with both high-dose and standard-dose amoxicillin. Based on the available evidence, tegoprazan 50 mg bd in combination with high-dose amoxicillin (750-1,000 mg three times daily) administered over 14 days represents the most consistently efficacious TADT regimen for treatment-naive patients. However, definitive dosing recommendations cannot be established from the present analysis alone, given the inter-study variability in dosing protocols. Dedicated, adequately powered, multi-phase clinical trials, including dose-optimisation and dose-ranging studies, are warranted to establish a standardised TADT regimen, evaluate its performance across geographically and genetically diverse populations, and assess its durability in the context of evolving antibiotic resistance patterns.

Strengths and Limitations

This study has several notable strengths. It is the first NMA to simultaneously compare TADT, BQT, and TBQT within a single network, enabling indirect comparisons not previously achievable through conventional pairwise meta-analysis. Consistency testing demonstrated acceptable coherence between direct and indirect evidence for all three outcomes, supporting the validity of the network estimates. The inclusion of both RCTs and a high-quality observational study broadens the evidence base.

Several limitations warrant acknowledgement. The modest number of included studies and sparse network for certain comparisons increases reliance on indirect evidence, which carries inherent uncertainty. Statistical heterogeneity arising from variability in tegoprazan dosing, amoxicillin intensity, and treatment duration was addressed through a random effects model, although residual heterogeneity may influence pooled estimates. The predominance of East Asian study populations limits generalisability to Western settings where resistance epidemiology and demographics differ. Heterogeneity in BQT antibiotic composition across studies may further affect comparative estimates. The absence of individual susceptibility testing data precluded resistance-stratified analysis. Publication bias cannot be excluded given the small number of eligible studies. Finally, inconsistent adverse event reporting necessitated a composite tolerability endpoint, obscuring granular differences between regimens. Future multicentre RCTs incorporating standardised regimens and prospective susceptibility testing across diverse populations are warranted.

## Conclusions

This network meta-analysis suggests that 14-day TADT, BQT, and TBQT achieve broadly comparable *H. pylori* eradication efficacy and treatment compliance as first-line strategies, although these findings should be interpreted in the context of a modest evidence base. TADT appeared associated with a lower risk of combined adverse events compared to BQT, indicating that it may represent a better-tolerated alternative, particularly for patients at heightened risk of antibiotic-associated toxicity or where simplified regimens are preferred. TBQT may offer an intermediate tolerability option potentially relevant in high-resistance settings. These findings provide preliminary network-level evidence supporting consideration of tegoprazan-based regimens as first-line options in future *H. pylori* eradication guideline updates. However, given the limited number of included studies, significant heterogeneity, and predominance of East Asian populations, large-scale multicentre RCTs across diverse geographic settings are necessary before definitive clinical recommendations can be established.

## Appendices

Appendix A: Database search strategies

PubMed/MEDLINE

("Helicobacter pylori"[MeSH] OR "H. pylori"[tiab] OR "Helicobacter pylori"[tiab] OR "Campylobacter pylori"[tiab]) AND ("tegoprazan"[tiab] OR "potassium-competitive acid blocker"[tiab] OR "P-CAB"[tiab] OR "amoxicillin"[tiab] OR "dual therapy"[tiab] OR "bismuth quadruple therapy"[tiab] OR "bismuth-containing quadruple therapy"[tiab] OR "quadruple therapy"[tiab] OR "tegoprazan-based quadruple therapy"[tiab]) AND ("randomized controlled trial"[pt] OR "randomised controlled trial"[tiab] OR "RCT"[tiab] OR "prospective"[tiab] OR "retrospective"[tiab] OR "cohort"[tiab] OR "observational study"[tiab] OR "eradication"[tiab] OR "adverse events"[tiab] OR "compliance"[tiab] OR "adherence"[tiab])

Embase

('Helicobacter pylori'/exp OR 'helicobacter pylori':ti,ab OR 'H. pylori':ti,ab OR 'campylobacter pylori':ti,ab) AND ('tegoprazan':ti,ab OR 'potassium competitive acid blocker':ti,ab OR 'P-CAB':ti,ab OR 'amoxicillin':ti,ab OR 'dual therapy':ti,ab OR 'bismuth quadruple therapy':ti,ab OR 'bismuth containing quadruple therapy':ti,ab OR 'quadruple therapy':ti,ab OR 'tegoprazan based quadruple therapy':ti,ab) AND ('randomized controlled trial':it OR 'randomised controlled trial':ti,ab OR 'prospective study':ti,ab OR 'retrospective study':ti,ab OR 'cohort':ti,ab OR 'observational study':ti,ab OR 'eradication':ti,ab OR 'adverse events':ti,ab OR 'compliance':ti,ab OR 'adherence':ti,ab)

Cochrane CENTRAL

("Helicobacter pylori" OR "H. pylori" OR "Campylobacter pylori") AND ("tegoprazan" OR "potassium-competitive acid blocker" OR "P-CAB" OR "amoxicillin" OR "dual therapy" OR "bismuth quadruple therapy" OR "bismuth-containing quadruple therapy" OR "quadruple therapy" OR "tegoprazan-based quadruple therapy") AND ("randomised controlled trial" OR "randomized controlled trial" OR "RCT" OR "clinical trial" OR "prospective" OR "cohort" OR "eradication" OR "adverse events" OR "compliance" OR "adherence")

Web of Science

TS=("Helicobacter pylori" OR "H. pylori" OR "Campylobacter pylori") AND TS=("tegoprazan" OR "potassium-competitive acid blocker" OR "P-CAB" OR "amoxicillin" OR "dual therapy" OR "bismuth quadruple therapy" OR "bismuth-containing quadruple therapy" OR "quadruple therapy" OR "tegoprazan-based quadruple therapy") AND TS=("randomized controlled trial" OR "randomised controlled trial" OR "RCT" OR "prospective" OR "retrospective" OR "cohort" OR "observational" OR "eradication" OR "adverse events" OR "compliance" OR "adherence")

Scopus

TITLE-ABS-KEY("Helicobacter pylori" OR "H. pylori" OR "Campylobacter pylori") AND TITLE-ABS-KEY("tegoprazan" OR "potassium-competitive acid blocker" OR "P-CAB" OR "amoxicillin" OR "dual therapy" OR "bismuth quadruple therapy" OR "bismuth-containing quadruple therapy" OR "quadruple therapy" OR "tegoprazan-based quadruple therapy") AND TITLE-ABS-KEY("randomized controlled trial" OR "randomised controlled trial" OR "RCT" OR "prospective" OR "retrospective" OR "cohort" OR "observational study" OR "eradication" OR "adverse events" OR "compliance" OR "adherence")

## References

[1] Hu Y, Xu X, He C (2025). Tegoprazan and low- or high-dose amoxicillin dual therapy versus bismuth-containing quadruple therapy for Helicobacter pylori eradication (TREAT): protocol for a multicenter, open-label, non-inferiority, randomized controlled trial. Ther Adv Gastroenterol.

[2] Chey WD, Howden CW, Moss SF, Morgan DR, Greer KB, Grover S, Shah SC (2024). ACG clinical guideline: treatment of Helicobacter pylori infection. Am J Gastroenterol.

[3] Kong Q, Mirza IA, Zhang X (2024). Fourteen-day tegoprazan-amoxicillin dual therapy as the first-line treatment of Helicobacter pylori infection (SHARE2301): a multicenter, noninferiority, randomized clinical trial. Helicobacter.

[4] Malfertheiner P, Camargo MC, El-Omar E (2023). Helicobacter pylori infection. Nat Rev Dis Primers.

[5] Jin T, Wu W, Zhang L, Xuan H, Zhang H, Zhong L (2025). The efficacy and safety of vonoprazan and tegoprazan in Helicobacter pylori eradication: a comprehensive systematic review and meta-analysis of randomized controlled trials. Ther Adv Gastroenterol.

[6] Cheng J, Zhao X, Fan C (2025). Tegoprazan dual and quadruple therapy for Helicobacter pylori eradication: a prospective, randomized controlled trial in Beijing, China. Front Med (Lausanne).

[7] Purja S, Lee Y, Kim E (2025). Tegoprazan as first- and second-line therapy for eradication of Helicobacter pylori infection: a systematic review and meta-analysis. J Dig Dis.

[8] Kim JS, Ko W, Chung JW, Kim TH (2023). Efficacy of tegoprazan-based bismuth quadruple therapy compared with bismuth quadruple therapy for Helicobacter pylori infection: a randomized, double-blind, active-controlled study. Helicobacter.

[9] Chen YC, Malfertheiner P, Yu HT (2024). Global prevalence of Helicobacter pylori infection and incidence of gastric cancer between 1980 and 2022. Gastroenterology.

[10] Sterne JA, Savović J, Page MJ (2019). RoB 2: a revised tool for assessing risk of bias in randomised trials. BMJ.

[11] Wells GA, Shea BJ, O'Connell D (2000). The Newcastle-Ottawa Scale (NOS) for Assessing the Quality of Non-randomized Studies in Meta-Analysis. Ottawa Hospital Research Institute.

[12] Fan Y, Li C, Zhang W (2026). Fourteen-and ten-day tegoprazan-amoxicillin dual therapy vs. bismuth quadruple therapy for Helicobacter pylori eradication-a noninferiority, multicenter, randomized controlled trial. J Gastroenterol Hepatol.

[13] Kim JY, Lee SY, Kim H, Kim JH, Sung IK, Park HS (2021). Efficacy of seven-day potassium-competitive acid blocker-based first-line Helicobacter pylori eradication therapy administered with bismuth. Yonsei Med J.

[14] Lin X, Huang H, Liu Y (2024). Tegoprazan-amoxicillin dual therapy for Helicobacter pylori eradication: a prospective, randomized, multicenter study in Fujian, China. Helicobacter.

[15] Liu HN, Wang R, Cao Y (2024). Comparison of the efficacy between the dual therapy of tegoprazan and the quadruple therapy of tegoprazan: a randomized controlled multicenter study. Clin Transl Gastroenterol.

[16] Song Z, Wang W, Li P (2025). Tegoprazan-based versus esomeprazole-based triple therapy plus bismuth for first-line Helicobacter pylori eradication: a nationwide, multicenter, double-blind, double-dummy, randomized controlled trial. Chin Med J (Engl).

[17] Yang J, Zhang X, Yang L (2025). Comparison of tegoprazan-based high-dose dual therapy versus bismuth-containing quadruple therapy for Helicobacter pylori eradication: a prospective, multicentre, randomised controlled trial in Gansu Province, a high-resistance region of China. Ther Adv Gastroenterol.

[18] Malfertheiner P, Megraud F, Rokkas T (2022). Management of Helicobacter pylori infection: the Maastricht VI/Florence consensus report. Gut.

[19] Scarpignato C, Hunt RH (2024). Potassium-competitive acid blockers: current clinical use and future developments. Curr Gastroenterol Rep.

[20] Salanti G, Ades AE, Ioannidis JP (2011). Graphical methods and numerical summaries for presenting results from multiple-treatment meta-analysis: an overview and tutorial. J Clin Epidemiol.

[21] Zhang JY, Chen JH, Huang YL, Li J, Xu D, Xu Z, Lei XY (2025). Fourteen-day vonoprazan-amoxicillin dual therapy versus 14-day bismuth-based quadruple therapy for Helicobacter pylori treatment: a randomized clinical trial. Ther Adv Gastroenterol.

[22] Chen X, Wang Y, Dong Y, Yang J, Xie B, Zhang D (2025). Helicobacter pylori eradication: why recurrence risk should not dictate treatment decisions. Ther Clin Risk Manag.

[23] Hong TC, El-Omar EM, Kuo YT (2024). Primary antibiotic resistance of Helicobacter pylori in the Asia-Pacific region between 1990 and 2022: an updated systematic review and meta-analysis. Lancet Gastroenterol Hepatol.

[24] Savoldi A, Carrara E, Graham DY, Conti M, Tacconelli E (2018). Prevalence of antibiotic resistance in Helicobacter pylori: a systematic review and meta-analysis in World Health Organization regions. Gastroenterology.

[25] Graham DY (2015). Helicobacter pylori update: gastric cancer, reliable therapy, and possible benefits. Gastroenterology.

